# Glutathione Levels and Susceptibility to Chemically Induced Injury in Two Human Prostate Cancer Cell Lines

**DOI:** 10.3390/molecules200610399

**Published:** 2015-06-05

**Authors:** Lawrence H. Lash, David A. Putt, Adam D. Jankovich

**Affiliations:** Department of Pharmacology, Wayne State University School of Medicine, Detroit, MI 48201, USA; E-Mails: dputt5@yahoo.com (D.A.P.); adajanko@umich.edu (A.D.J.)

**Keywords:** glutathione, prostate cancer cells, oxidative stress, apoptosis, stress response, mitochondria

## Abstract

More aggressive prostate cancer cells (PCCs) are often resistant to chemotherapy. Differences exist in redox status and mitochondrial metabolism that may help explain this phenomenon. Two human PCC lines, PC-3 cells (more aggressive) and LNCaP cells (less aggressive), were compared with regard to cellular glutathione (GSH) levels, susceptibility to either oxidants or GSH depletors, and expression of several proteins involved in apoptosis and stress response to test the hypothesis that more aggressive PCCs exhibit higher GSH concentrations and are relatively resistant to cytotoxicity. PC-3 cells exhibited 4.2-fold higher GSH concentration than LNCaP cells but only modest differences in acute cytotoxicity were observed at certain time points. However, only LNCaP cells underwent diamide-induced apoptosis. PC-3 cells exhibited higher levels of Bax and caspase-8 cleavage product but lower levels of Bcl-2 than LNCaP cells. However, LNCaP cells exhibited higher expression of Fas receptor (FasR) but also higher levels of several stress response and antioxidant proteins than PC-3 cells. LNCaP cells also exhibited higher levels of several mitochondrial antioxidant systems, suggesting a compensatory response. Thus, significant differences in redox status and expression of proteins involved in apoptosis and stress response may contribute to PCC aggressiveness.

## 1. Introduction

Prostate cancer is the most prevalent cancer afflicting adult males in the U.S. and is the second leading cause of cancer-related deaths in American men as of 2008 [1]. Much evidence has implicated the involvement of oxidative stress and modulation of cellular redox status as major mechanisms associated with the development and progression of prostate cancer [2,3,4,5,6,7,8,9,10,11]. Two parameters that have received particular attention have been cellular and mitochondrial glutathione (GSH) status and mitochondrial energetics. For example, Chaiswing *et al*. [4] compared redox state of two human prostate carcinoma cell (PCC) lines, LNCaP and PC-3 cells, and reported that levels of lipid peroxidation byproducts, reactive oxygen species (ROS), and reactive nitrogen species (RNS) were higher in LNCaP cells whereas medium concentrations of GSH and glutathione disulfide (GSSG) were much higher in PC-3 cells. Moreover, they also observed that thiol redox status and activities and expression of several antioxidant enzymes exhibited distinct patterns in the two PCC lines at different growth phases, suggesting that modulation of thiol redox status may be useful as a therapeutic tool to modify PCC proliferation and tumor aggressiveness.

Several studies have provided the basis for what Costello and Franklin [12] have called the “bioenergetic theory of prostate malignancy”. According to this concept, mitochondrial metabolism is altered in malignancy so that in contrast to normal or benign hyperplastic prostate epithelial cells, which accumulate citrate due to low activity of mitochondrial aconitase, malignant prostate epithelial cells have become citrate-oxidizing cells and exhibit low amounts of citrate due to high activity of the enzyme. The higher rate of mitochondrial substrate metabolism also helps explain the increased levels of ROS associated with the malignant and metastatic PCCs. It is also well established that defects in the mitochondrial genome lead to mitochondrial dysfunction and are characteristic of numerous types of cancer, including prostate cancer [13]. Additionally, experimental activation of the mitochondrial apoptotic pathway in PCC lines by various chemicals or hormones is another therapeutic approach that emphasizes the importance of the mitochondria in prostate cancer [14,15,16,17,18,19].

The foregoing discussion illustrates the importance of mitochondrial function and redox status (one major component being GSH) in determining metastatic aggressiveness and sensitivity to chemotherapeutic agents of PCCs, and provides the rationale for a focus on mitochondrial GSH (mtGSH) as a potential therapeutic target in prostate cancer. The present work characterizes some basic properties of redox status and susceptibility to chemical toxicants of two PCC lines, PC-3 and LNCaP cells, to test the hypothesis that differences in GSH status and expression of proteins involved in regulation of apoptosis and stress response are correlated with differences in susceptibility to cytotoxic chemicals or chemotherapeutic agents. The overall objective of these studies, therefore, is to provide an initial validation of the principle that cellular GSH level in cancer cells may be a target for therapeutic manipulation. Some portions of this study were presented in abstract form at the 2014 Society of Toxicology [20] and Experimental Biology [21] meetings.

## 2. Results

### 2.1. GSH Concentrations and Oxidant-Induced GSH Oxidation in PC-3 and LNCaP Cells

**Figure 1:**
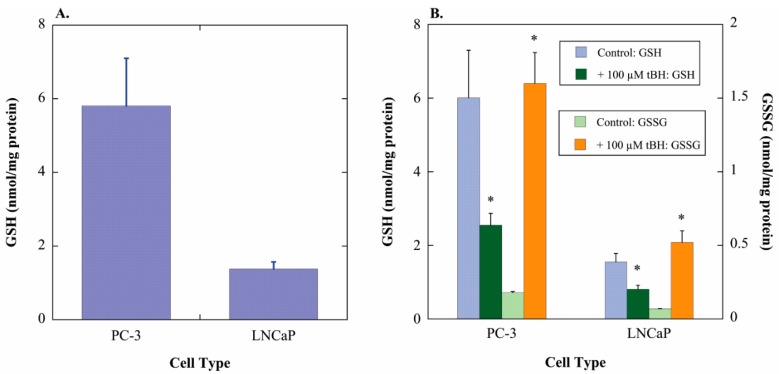
GSH content and oxidant-induced GSH oxidation in PC-3 and LNCaP cells. (**A**) GSH was measured in total cell extracts by the GSH Glo™ Glutathione Assay kit, which is a luminescence-based assay coupling the GSH *S*-transferase (GST)-catalyzed conversion of a luciferin derivative to luciferin, which is then metabolized by the ATP-dependent firefly luciferase to emit light, which is then read in a plate reader in the luminescence setting. GSH content was calculated from a GSH standard curve. Results are means ± SEM of measurements from 3 separate cultures; (**B**) Cells were incubated for 1 h with either cell culture medium (=Control) or 100 µM *tert*-butyl hydroperoxide (tBH). Concentrations of GSH and GSSG in total cell extracts were then measured by use of Ellman’s reagent with or without 2-vinylpyridine, respectively, and measurement of A_412_. GSH and GSSG contents were calculated from a GSH standard curve. Results are means ± SEM of measurements from three separate cultures. * Significantly different (*p* < 0.05) from the corresponding control.

To validate the hypothesis that more aggressive PCC lines have higher concentrations of GSH and that this is associated with greater growth potential, GSH contents of PC-3 cells (highly aggressive) and LNCaP cells (modestly aggressive) were measured. The more aggressive cell line, PC-3 cells, exhibited 4.2-fold higher content of GSH (Figure 1A), consistent with the proposed hypothesis and the more rapid growth rate of PC-3 cells. The two cell lines were then incubated for 1 h with either cell culture medium (=Control) or 100 µM *tert*-butyl hydroperoxide (tBH) to assess oxidation of GSH to glutathione disulfide (GSSG) (Figure 1B). PC-3 and LNCaP cells exhibited similar GSH/GSSG ratios in both the absence (16.7 and 11.1, respectively) and presence of oxidant (0.80 and 0.78, respectively). Thus, the extent of GSH loss was similar in both cell lines and was accounted for by increases in GSSG.

**Figure 2:**
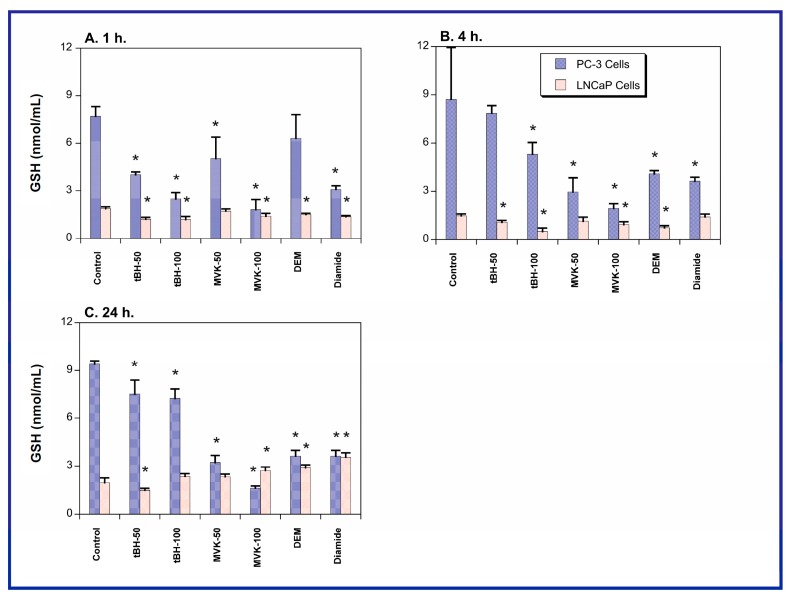
GSH content in PC-3 and LNCaP cells. GSH was measured in total cell extracts by use of Ellman’s reagent and measurement of A_412_. GSH content was calculated from a GSH standard curve. Results are means ± SEM of measurements from six separate cultures. * Significantly different (*p* < 0.05) from the corresponding control.

To further assess the GSH pool in the two cell lines, each cell line was treated for up to 24 h with either cell culture medium (=Control) or various toxicants and GSH concentrations were measured at 1, 4, and 24 h (Figure 2). The toxicants used to probe responses to oxidants or GSH-depleting agents included tBH, which causes lipid peroxidation and GSH oxidation, methyl vinyl ketone (MVK), which is a direct-acting alkylating agent that depletes GSH, and diethyl maleate (DEM) and diamide, both of which are electrophiles that deplete GSH by forming GSH *S*-conjugates. tBH and MVK were added at 50 µM or 100 µM and DEM and diamide were each added at 250 µM. Both cell lines exhibited generally similar degrees of GSH depletion, except at the 100 µM concentration of tBH or MVK, where toxicant treatment of PC-3 cells reduced cellular GSH concentrations to levels close to those found in the LNCaP cells. Thus, it appears that susceptibility of the cellular GSH pool of the two cell lines to oxidants cannot explain the differences in their growth rates or sensitivity to chemotherapeutic agents.

### 2.2. Acute Cellular Necrosis and Apoptosis Induced by Toxicants in PC-3 and LNCaP Cells

**Figure 3:**
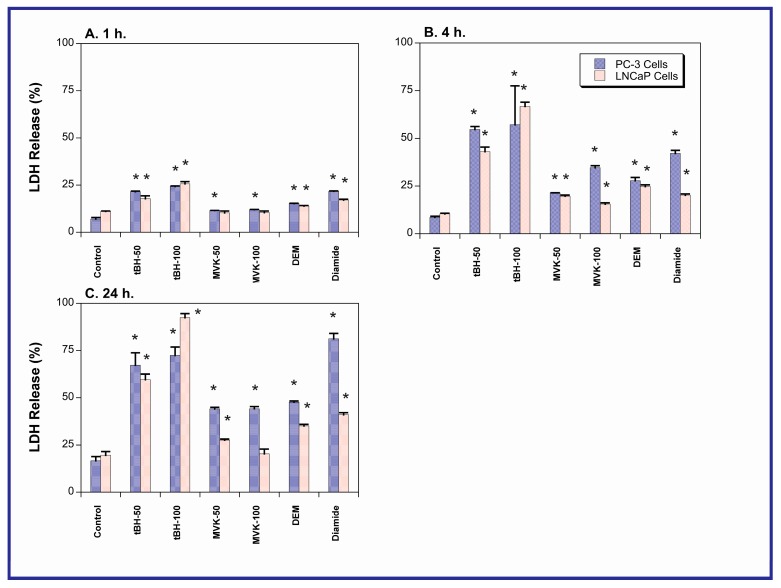
Chemically induced LDH release in PC-3 and LNCaP cells. Cells were grown to 80%–90% confluence in either F12 or RPMI media, respectively, supplemented with 10% FBS. Prior to experiments, media were removed and replaced with serum-free media. After 1-, 4-, or 24-h incubations with either tBH or MVK (10, 50, 100, 200 µM), or 250 µM of either DEM or diamide, LDH release was determined as NADH oxidation at 340 nm. Results are the means ± SEM of measurements from six cell cultures. * Significantly different (*p* < 0.05) from the corresponding control.

Acute cell death was determined with the lactate dehydrogenase (LDH) release assay by incubating PC-3 and LNCaP cells as above, with either culture medium (=Control), tBH, MVK, DEM, or diamide for 1, 4, or 24 h (Figure 3). In contrast to expectations, LNCaP cells were only more sensitive to some of the toxicants at a few time points. At the 24-h time point, PC-3 cells were actually noticeably more sensitive to MVK, DEM, and diamide than were the LNCaP cells.

**Figure 4:**
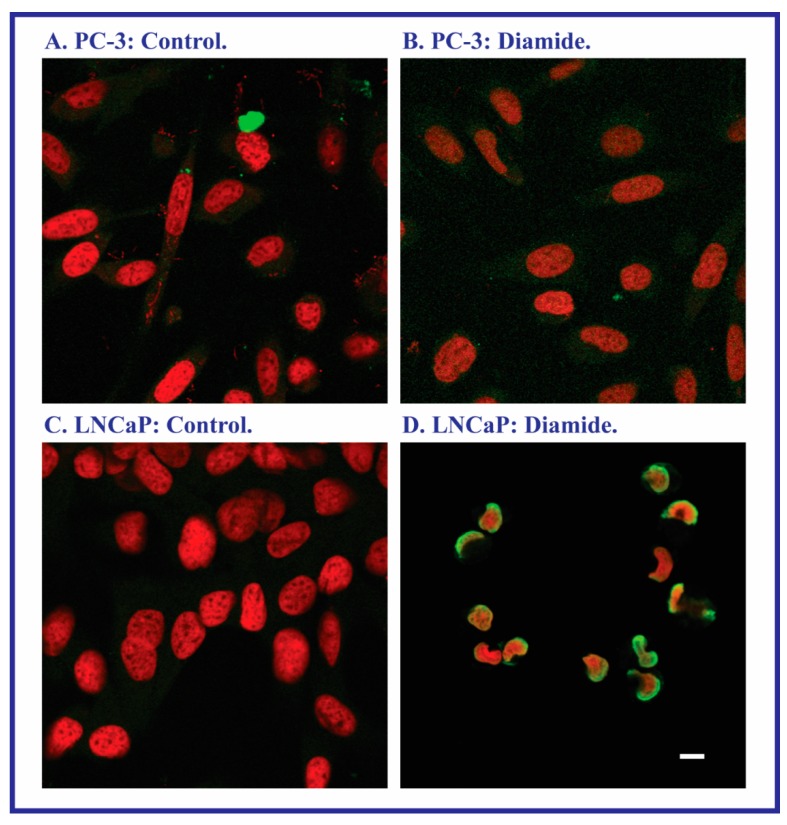
Sensitivity of PC-3 and LNCaP cells to diamide-induced apoptosis as assessed by the TUNEL assay. DNA fragmentation was assayed with the ApoBrdU TUNEL Assay Kit from Invitrogen (Carlsbad, CA, USA), using confocal microscopy. Cells were grown on 35 mm dishes. After incubation for 24 h with either media (=Control) or 250 µM diamide, cells were fixed in paraformaldehyde and 70% ethanol. DNA strand breaks were detected by an Alexa Fluor 488 dye-labeled anti-BrdU antibody and the resultant green nuclear fluorescence in contrast to the red fluorescence generated from nuclear propidium iodide staining. Bar = 5 µm.

Although LDH release may reflect cell death due to both necrosis and apoptosis, assessment of apoptosis by multiple assays suggested that the observed LDH release was primarily due to necrosis. Apoptosis was assessed by measurement of tBH- or MVK-induced activation of caspase-3/7 and caspase-8 and DNA fragmentation by agarose gel electrophoresis (data not shown). For all three assays, no evidence of oxidant-induced apoptosis was obtained. In contrast, the terminal deoxynucleotidyl transferase dUTP nick end-labeling (TUNEL) assay was also used to assess DNA damage as an indicator of apoptosis. With the exception of diamide, none of the toxicants produced a positive response. In the case of diamide, however, apoptosis was clearly evident in LNCaP cells but not in PC-3 cells (Figure 4).

### 2.3. Expression of Apoptosis, Stress Response, and Mitochondrial Proteins in PC-3 and LNCaP Cells

Results from measurements of GSH concentrations, responses of the cellular GSH pools to toxicants, and acute cytotoxic responses of the two cell lines to toxicants suggest that differences between the two cell lines in aggressiveness and sensitivity to chemotherapeutic agents cannot be simply explained by the approximately fourfold difference in total GSH contents. Rather, more complex responses to other processes likely underlie the pathophysiological differences in the two cell lines.

**Figure 5:**
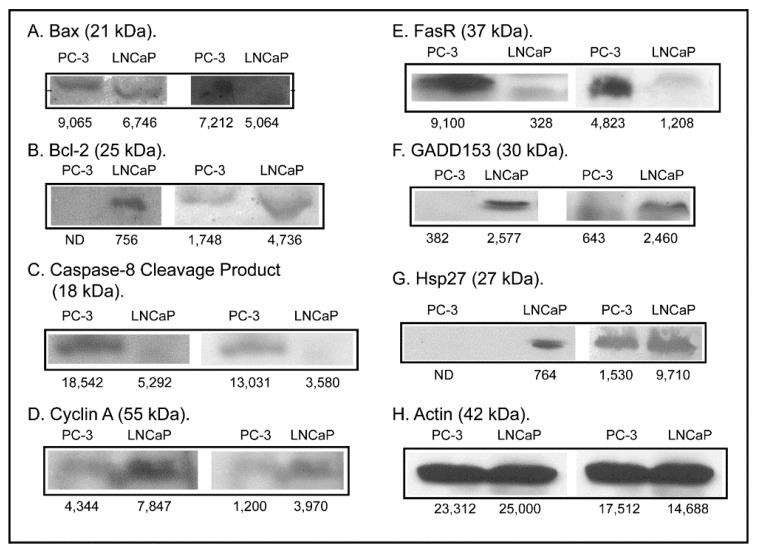
Western blot analysis of selected proteins regulating apoptosis, cell growth, and stress response. Protein (100 µg) from total cell extracts of PC-3 and LNCaP cells were loaded onto 10%, 12%, or 15% SDS polyacrylamide gels. After electroblotting of protein onto nitrocellulose paper, blots were blocked for 1 h in 5% milk powder solution and incubated overnight with primary antibody. Blots were washed 3× with TTBS and incubated with appropriate secondary antibody conjugated to alkaline phosphatase (Jackson ImmunoResearch, West Grove, PA, USA) or horseradish peroxidase, for 1 h. Blots were washed 3–6× in TTBS and then assayed for color development using BCIP/NBT as substrates (Promega, Madison, WI, USA) or using the Pierce ECL Western Blotting Substrate Kit. β-Actin was used as a housekeeping protein. Band density for samples was determined with GelEval 1.3.7 for Mac OS X. Results are from two sets of cell cultures for each cell line.

To investigate the hypothesis that differences in other processes more complex than GSH status underlie the functional differences between the two cell lines, expression of several proteins known to play significant roles in regulation of apoptosis and stress response was assessed (Figure 5). Unlike the modest or lack of differences in acute cellular necrosis and GSH depletion, expression of two pro-apoptotic proteins, Bax and caspase-8 cleavage product, was markedly higher in PC-3 cells (11.1- and 3.4-fold, respectively, adjusting for actin expression) whereas expression of Fas receptor (FasR) was 2.9-fold higher in LNCaP cells and that of the anti-apoptotic protein Bcl-2 was clearly visible in LNCaP cells but was undetectable in PC-3 cells. Expression of three stress response proteins was also assessed. While expression of cyclin A was only modestly higher in LNCaP cells (1.3-fold, adjusted for actin), expression of Growth Arrest and DNA Damage 153 (GADD153) and heat shock protein 27 (Hsp27) was markedly higher in LNCaP cells (26.5- and 54.7-fold, respectively, adjusting for actin). These protein expression differences suggest that PC-3 cells should be more sensitive to apoptosis by the intrinsic or mitochondrial pathway and that LNCaP cells possess higher levels of cytoprotective proteins such as GADD153 and Hsp27. 

**Figure 6:**
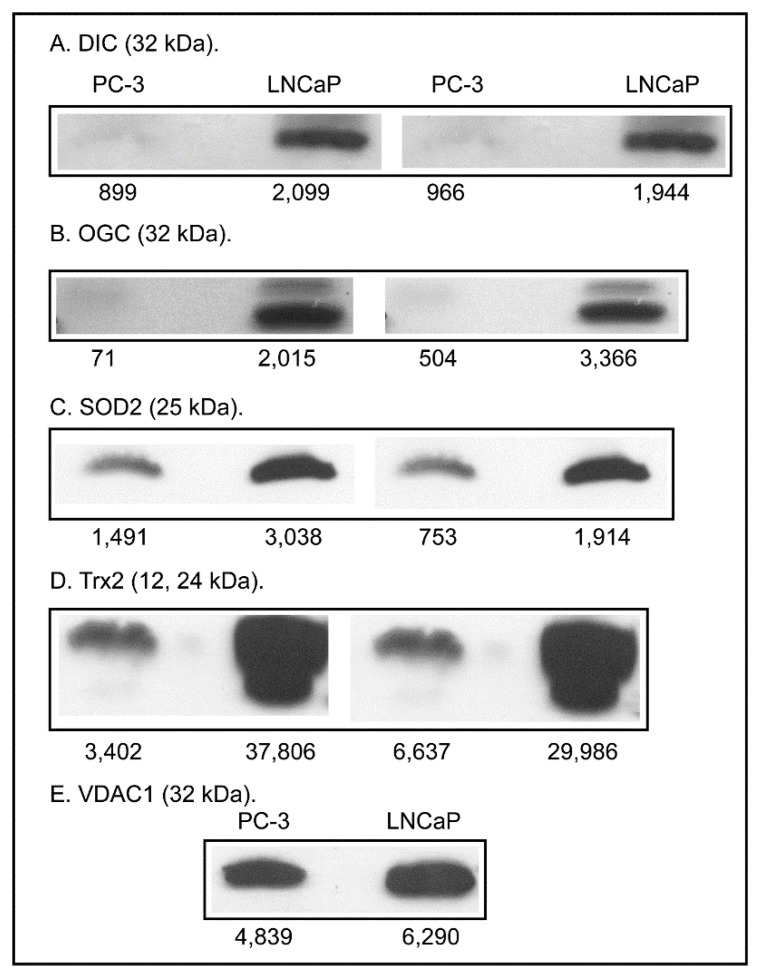
Western blot analysis of selected proteins of mitochondrial redox status*.* Samples were processed as described in the legend to Figure 5. VDAC was used as a housekeeping protein. Band density for samples was determined with GelEval 1.3.7 for Mac OS X. Results are from two sets of cell cultures for each cell line, except for VDAC blots, which were from a single set of cell cultures.

This contrasts with the observations on sensitivity of the two cell lines to tBH-, MVK-, DEM-, and diamide-induced LDH release. Higher levels of these cytoprotective proteins may be a compensatory response to the low GSH concentrations characteristic of this cell line. The higher expression of FasR in LNCaP cells, however, suggests that these cells are more susceptible to apoptosis by the extrinsic pathway. Although the distinct mitochondrial pool of GSH was not separately analyzed in this study, differences in mitochondrial function may be critical to the proliferative properties of the two cell lines, as suggested by the “bioenergetics theory of prostate malignancy” [12]. As a first step in assessing the involvement of mitochondria in differentiating the aggressiveness and growth of these two PCC lines, expression of two mitochondrial inner membrane carriers involved in the uptake of GSH and two important antioxidant proteins was determined (Figure 6).

Renal mitochondria [22], like hepatic mitochondria [23], do not appear to synthesize GSH *de novo* but must transport GSH from the cytoplasm into the mitochondrial matrix. The transport is mediated by either the dicarboxylate carrier (DCC; *Slc25a10*) or 2-oxoglutarate carrier (OGC; *Slc25a11*) [24,25]. Again, in what would appear to be a compensatory response, expression of both the DCC and OGC was much higher in LNCaP cells than in PC-3 cells (4.5- and 2.1-fold higher, respectively, adjusted for expression of the voltage-dependent anion channel [VDAC]). Similarly, expression of two enzymes important for antioxidant defense, superoxide dismutase 2 (SOD2) and thioredoxin 2 (Trx2), were modestly higher in LNCaP cells than in PC-3 cells (1.5- and 1.7-fold higher, respectively, adjusted for expression of VDAC).

## 3. Discussion

PC-3 and LNCaP cells were studied as models of a highly aggressive and less aggressive PCC line, respectively. PC-3 cells are adherent, grow rapidly, form clusters in soft agar, can be adapted to suspension growth, and are highly tumorigenic. In contrast, LNCaP cells grow relatively slowly, are adherent, grow as single cells or loosely attached clusters, and are also tumorigenic, although less so than PC-3 cells. A positive relationship between tumor cell growth and cellular GSH levels in both PCCs [26,27] and other types of cancer cells [28,29] has been known for many years. A previous study by Chaiswing *et al.* [4] specifically comparing PC-3 and LNCaP cells showed that the cellular concentration of GSH was markedly higher in PC-3 cells than in LNCaP cells and that this difference was associated with higher levels of ROS, RNS, and cytotoxic byproducts of oxidative injury in LNCaP cells. Based on these findings, we hypothesized that the higher levels of GSH and other antioxidants in PC-3 cells exist and are associated with the differential susceptibility of the two cell lines to toxicants and chemotherapeutic agents.

Results from the present study confirmed the existence of a markedly higher concentration of GSH in PC-3 cells as compared to LNCaP cells. The two cell lines exhibited similar GSH/GSSG ratios and their GSH pools were oxidized to GSSG to a similar extent after inhibition with the oxidant tBH. Thus, there do not appear to be differences in the ability of the two cell lines to maintain an appropriate GSH/GSSG ratio. Other processes that are known to regulate cellular GSH status include activity and/or expression of glutamate-cysteine ligase or GSH synthase, availability of l-cysteine, which is rate-limiting for GSH synthesis, and activity and/or expression of plasma membrane transporters. Further study to measure these parameters is needed to identify the mechanism(s) by which different PCC lines regulate their GSH supply and redox status.

What is the impact of such markedly different cellular concentrations of GSH on cellular function and susceptibility to toxic chemicals? How are these differences transmitted to the cell? Recent reviews by Ortega *et al*. [30] and Circu and Aw [31] provide some insight into the underlying signaling mechanisms that translate differences in GSH levels and redox status into functional differences. For example, it is clear that thiol (primarily GSH) oxidation is a causal factor in mitochondrial-based cell death and that many GSH-dependent and other antioxidant enzymes are regulated by the redox-sensitive transcription factor NF-E2 p45-related factor-2 (Nrf2) [30]. GSH is also involved in several apoptosis signaling pathways, such as the mitogen-activated protein kinase (MAPK) and Fas ligand/Fas receptor (FasL/FasR) pathways [31]. Further analysis of these pathways may help identify therapeutic targets in PCCs and other cancer cells that can improve the efficacy of chemotherapy.

Despite the expectation that LNCaP cells would be significantly more susceptible than PC-3 cells to oxidants and thiol-alkylating agents (tBH, MVK, DEM, diamide), only modest differences in LDH release at selected time points were observed. Moreover, under some conditions PC-3 cells exhibited more LDH release than LNCaP cells. Additionally, both cell lines exhibited similar degrees of GSH depletion when exposed to the four toxicants. While this might suggest little difference in susceptibility to oxidants such as those studied here, another possible interpretation is that the two cell lines possess a different capacity to function with lower GSH concentrations. The rationale for choosing 1, 4, and 24 h as the exposure times is based on the desire to observe cytotoxicity at both very early times and after a long enough exposure time that would enable activation of key cellular responses and signaling pathways.

Increases in LDH release are typically observed in necrotic cell injury, when there is rupture of cellular plasma membranes. However, they may also be observed in late-apoptosis. When apoptosis was specifically assessed by several assays, only the TUNEL assay demonstrated the occurrence of apoptosis. Of the four toxicants studied, only diamide produced a positive response in LNCaP cells and no effects were observed in PC-3 cells. Examination of the expression of pro- and anti-apoptotic proteins in the two cell lines and the absence of significant caspase activation suggest that diamide-induced apoptosis in LNCaP cells probably occurs by the FasR pathway. Although PC-3 cells exhibited higher expression of Bax and caspase-8 cleavage product and lower expression of Bcl-2, no apoptosis due to any of the toxicants was observed in these cells. This resistance is likely due to the higher GSH concentration. PC-3 cells are well-known to be resistant to apoptosis. Gumelec *et al.* [32] studied cisplatin-induced apoptosis in three human PCC lines, including PC-3 cells. These authors concluded that differences in cell cycle regulation were at least partially responsible for the resistance to cisplatin. They also found that the absence of p53 and lower levels of Bax contributed to the cytoresistance of PC-3 cells as compared to the other two cell lines. We also confirmed the absence of p53 in PC-3 cells, similar to Skjoth and Issinger [33], whereas p53 was readily detectable in LNCaP cells (data not shown).

Such observations, along with the fact that there was no direct correlation between GSH concentrations and susceptibility to toxicants, led us to hypothesize that more complex differences between the two cell lines, potentially involving signaling pathways and key proteins involved in stress response and redox regulation, were likely the underlying mechanisms for some of the differences between the two cell lines. Besides the differences in Bax and Bcl-2 noted above, protein expression levels of cyclin A, GADD153, and Hsp27 were higher in LNCaP cells, with the latter two proteins being markedly higher. We interpret this as a compensatory response in LNCaP cells due to their relatively low GSH concentration. Although induction of apoptosis did not appear to be the major mechanism of cytotoxicity with the chemicals and conditions used in the present study, further investigation of apoptotic signaling pathways in the two cell lines may provide further insight into factors that determine chemotherapeutic efficacy.

Although we did not specifically quantify the mtGSH pool due to technical difficulties in obtaining good quality mitochondria, it is likely that this pool and other redox components in the mitochondria may be the critical determinants of PCC growth and aggressiveness. Measurements of protein expression of the two mtGSH transporters, the OGC and DIC, and of two important antioxidant enzymes in mitochondria, SOD2 and Trx2, showed all to be significantly higher (between 1.5- and 4.5-fold) in LNCaP cells, again suggesting a compensatory response. While expression of the two mtGSH transporters was increased, targeting of these proteins may provide for a novel therapeutic target to enhance the sensitivity of PCCs to chemotherapeutic agents. The situation here is the opposite of what we were trying to do with two chronic pathological states affecting renal mitochondria, namely diabetic nephropathy [34,35] and compensatory renal hypertrophy [36,37]. In both of these cases, there is a modest compensatory increase in expression and activity of mtGSH transporters but the renal cells are still in a state of oxidative stress and enhanced susceptibility to oxidant-induced injury. The goal in diabetic nephropathy or compensatory renal hypertrophy is to further overexpress the mtGSH transporters to provide for sustained increases in mtGSH concentration to counteract and protect against the oxidative stress. For prostate cancer, however, specific knockdown of these carriers may be a means to eliminate a key component of the compensatory response and make the PCCs more susceptible to chemotherapeutic agents. Clearly more investigation specifically targeted on the mtGSH pool is warranted.

## 4. Experimental Section

### 4.1. Cell Culture and Incubations

PC-3 (cat. no. CRL-1435) and LNCaP (cat. no. CRL-1740) cells were purchased from the American Type Culture Collection (ATCC; Manassas, VA, USA). PC-3 cells were grown in F12 media supplemented with 10% fetal bovine serum. LNCaP cells were grown in RPMI 1640 media supplemented with 10% fetal bovine serum. Cells were grown on T-75 tissue culture flasks (11 mL of 1 × 10^6^ cells/mL) for western blots or on 24-well plates (0.5 mL per well of 1 × 10^6^ cells/mL) for LDH release or GSH assays. Both PC-3 and LNCaP cells were treated with varying concentrations of tBH (10, 50, and 100 µM), MVK (10, 50, and 100 µM), diethyl maleate (250 µM) or diamide (250 µM) for varying lengths of time (1, 4, or 24 h).

### 4.2. GSH and GSSG Measurements

GSH was measured by either the GSH Glo^®^ Glutathione Assay Kit (Promega, Madison WI, USA) or by use of Ellman’s reagent [38], as indicated in the figure legends. For the GSH Glo^®^ Glutathione Assay Kit method, 50 µL of sample and 50 µL of GSH-Glo^®^ Reagent were incubated for 30 min in 24-well opaque microplates before adding 100 µL of Luciferin Detection agent. Following a 15-min incubation, the plates were read in a SpectraMax2 plate reader (Molecular Devices; Sunnyvale, CA, USA). GSH content was calculated from a standard curve created from a 5 mM GSH standard supplied by the manufacturer. For the Ellman’s reagent method, incubation of samples with 5,5′-dithiobis(2-nitrobenzoic acid) [DTNB] and measurement of the absorbance change at 412 nm was conducted as described in the original method [38]. GSSG was measured by the method of Griffith [39], which involves first trapping endogenous GSH with 2-vinylpyridine and then measuring GSSG as GSH-equivalents after reduction with DTNB.

### 4.3. LDH Release Cell Viability Assay

Cell viability was assessed by measuring the release of LDH into the medium following exposure to the various toxicants. Briefly, cells were grown on 24-well plates and following treatment with the toxicant, the cell lysate and the growth medium (either with or without serum) were assayed at 340 nm separately for 3 min for the oxidation of NADH in the presence of added pyruvate. The percentage of cell viability was calculated by dividing the slope obtained from the cell medium by the combined slopes of the total cell extract and cell medium and multiplying by 100.

### 4.4. TUNEL Assay

DNA fragmentation was assayed with the ApoBrdU TUNEL Assay Kit (Invitrogen; Carlsbad, CA, USA) using confocal microscopy. The cells were grown in 35-mm dishes, and following treatment were fixed in paraformaldehyde and 70% ethanol. DNA strand breaks were detected by an Alexa Fluor 488 dye-labeled anti-BrdU antibody and the resultant green nuclear fluorescence in contrast to the red fluorescence generated from nuclear propidium iodide staining.

### 4.5. Determination of Protein Expression by Western Blot Analyses

#### 4.5.1. Basic Sample Preparation and General Assay Information

Protein (100 µg) was loaded in the wells of 10%, 12% or 15% polyacrylamide gels. After electroblotting of protein onto nitrocellulose paper the blots were blocked for 1 h in 5% milk powder solution and incubated overnight with the primary antibody. The blots were washed three times with Tris-buffered saline containing Tween 20 (TTBS) and incubated with the appropriate secondary antibody conjugated to alkaline phosphatase (Jackson ImmunoResearch; West Grove, PA, USA) or horseradish peroxidase for 1 h. Blots were washed 3–6 times in TTBS and then assayed for color development using 5-bromo-4-chloro-3-indolyl phosphate/nitroblue tetrazolium (BCIP/NBT) as substrates (Promega) or exposed for visualization on autoradiography film (Denville Scientific; Metuchen, PA, USA) using the Pierce enhanced chemiluminescence (ECL) Western Blotting Substrate Kit (Pierce; Rockford, IL, USA). For all blots, the order of loading was: Lane 1 = PC-3 cells; Lane 2 = LNCaP cells; Lane 3 = molecular weight markers. Protein was measured using the bicinchoninic acid protein assay kit (Pierce) at a wavelength of 532 nm. A standard curve plot of bovine serum albumin concentration *vs.* absorbance was generated to determine sample protein concentration.

#### 4.5.2. Antibody Information

Antibody to actin, which was used as a housekeeping protein for analyses with total cell extracts, was a rabbit polyclonal antibody that recognizes the 42-kDa protein in a variety of species and tissues (Sigma Aldrich; St. Louis, MO, USA). Antibody to Bcl-2 was a mouse monoclonal antibody that recognizes human Bcl-2 (mw 24–26 kDa; CalBiochem/EMD Biosciences; Darmstaedt, Germany). Antibody to Bax was a rabbit polyclonal antibody that recognizes the monomeric human Bax protein (21 kDa) and the 44–50 kDa dimeric Bax protein (CalBiochem/EMD Biosciences). Antibody to cleaved caspase-8 (18 kDa fragment) and cyclin A were mouse monoclonal antibodies (Cell Signaling Technology; Danvers, MA, USA). Antibodies to the DIC and OGC were rabbit polyclonal antibodies (Abcam; Cambridge, MA, USA). Antibody to FasR was a rabbit polyclonal antibody that recognizes the human Fas receptor (mw 48 kDa; Santa Cruz Biotechnology, Santa Cruz, CA, USA). Antibody to GADD153 was a mouse monoclonal antibody raised against amino acids 1–168 of the full-length mouse GADD153 (mw 30 kDa; Santa Cruz Biotechnology). Antibody to Hsp27 was a mouse monoclonal antibody that recognizes human and monkey heat shock protein 27 (mw 27 kDa; StressGen, Victoria, BC, Canada).

### 4.6. Statistical Analysis

Results are expressed as means ± SEM. Densitometry of bands on western blots were calculated using GelEval 1.3.7 software for Mac OS X. Student’s *t*-test was performed to determine which means were significantly different from one another, using a two-tail probability of *p* < 0.05 as the criteria for significance.

## 5. Conclusions

In summary, the present work has confirmed the markedly higher concentration of total cellular GSH in the more aggressive PC-3 cells as compared to the less aggressive LNCaP cells. However, assessments of toxicant-induced GSH depletion or acute cytotoxicity (LDH release) did not show the expected greater susceptibility to toxicant-induced GSH depletion and cell death, although diamide-induced apoptosis was only detected in LNCaP cells. Examination of protein expression for several proteins involved in regulation of apoptosis and stress response showed that despite its relatively low GSH concentration, LNCaP cells exhibit complex compensatory responses involving relatively high expression of cytoprotective and antioxidant proteins and of mitochondrial proteins. Alterations in mitochondrial proteins may be a key to understanding what determines PCC growth, aggressiveness, and susceptibility to chemotherapeutic agents.
